# Advancements in Biomaterials for Bioengineering and Biotechnology

**DOI:** 10.3390/ijms25147840

**Published:** 2024-07-17

**Authors:** Alexander Tsouknidas

**Affiliations:** 1 Laboratory for Biomaterials and Computational Mechanics, Department of Mechanical Engineering, University of Western Macedonia, 50100 Kozani, Greece; atsouknidas@uowm.gr or atsouk@bu.edu; 2 Department of Restorative Sciences & Biomaterials, Henry M. Goldman School of Dental Medicine, Boston University, Boston, MA 02118, USA

Biomaterials, whether of biological or synthetic origin, have risen to the forefront of modern medical innovation since the early 2000s, transcending their traditional roles in orthopedic and dental applications, to encompass drug delivery systems, implantable biosensors, and templates for cellular growth and tissue regeneration.

The unprecedented advancements in both implantable and extracorporeal material-based therapeutics, were driven in part by our capacity to functionalize their surface properties, along with recent advances facilitating the manipulation of matter on the nanoscale. As a result, multifunctional biomaterials have received immense interest within the materials community, providing biomedical engineers, physicians, and other medical and biotechnology professionals with novel therapeutic approaches.

This Special Issue encapsulates the latest advancements in this dynamic field, showcasing diverse applications that converge to advance our understanding of biological systems.

The featured manuscripts exemplify the breadth and depth of contemporary biomaterials research. Molecular dynamics simulations shed light on the mechanical properties of hexagonal hydroxyapatite, illuminating the impact of pore defects on its structural integrity [1]. Another experimental investigation elucidates the physical and electrochemical properties of protic ionic liquids, laying the groundwork for their potential applications in various domains [2].

The synthesis and characterization of novel hybrid materials, such as fluorescent mesoporous silica-based compounds, underscore the ongoing quest to engineer biomaterials with tailored functionalities. These materials exhibit not only remarkable antioxidant activity but also promising antitumor properties, opening new avenues for cancer therapy [3]. In another study, mesoporous silica was used as a shell material for LiYF_4_ nanoplatforms to increase reactive oxygen species (ROS) production to achieve effectual photodynamic therapy, which exhibited prominent therapeutic efficacy [4].

Other hybrid materials (i.e., cross-linked hyaluronate with corticosteroids) were directly employed in animal models, showcasing the potential of biomaterials in the treatment of tendinopathy [5].

The intersection of biomaterials and regenerative medicine was explored through an innovative approach, 3D bioprinting, which holds immense promise for personalized medicine [6]. Although challenges remain in achieving anatomically realistic constructs with mature biological functions, the rapid progress in this field underscores its potential to revolutionize tissue engineering and drug discovery. Microfluidic organ-on-a-chip devices represent another frontier in biomaterials research, offering platforms for modeling complex organ systems with unprecedented fidelity. Microfluidic organ-on-a-chip devices have recently been leveraged, aiming, among other goals, to further our understanding of tissue engineering through advanced ex vivo models. Fueled by a diverse array of biomaterials and fabrication techniques, researchers are poised to determine the intricacies of human physiology and develop novel strategies for disease modeling and drug screening [7].

Lastly, the development of light-controlled activation strategies for platinum (IV) prodrugs exemplifies the convergence of materials science and therapeutic innovation. Harnessing the power of external stimuli, such as light, enables precise spatiotemporal control over drug release, minimizing off-target effects and enhancing therapeutic efficacy [8].

In closing, the collective efforts showcased in this Special Issue highlight the remarkable progress in the field of biomaterials for bioengineering and biotechnology. From fundamental studies elucidating mechanical properties to translational research aimed at clinical application, each contribution represents a step forward as we navigate the complexities of human health and disease.

I extend my gratitude to the authors, reviewers, and editorial team for their invaluable contributions, hoping that this Special Issue will serve as a source of inspiration and insight for researchers and practitioners alike, spurring further advancements at the intersection of materials science, biology, and medicine.
